# Congenital absence of inferior vena cava and thrombosis: a case report

**DOI:** 10.1186/1752-1947-2-46

**Published:** 2008-02-12

**Authors:** Javaid Iqbal, Eswarappa Nagaraju

**Affiliations:** 1 Dept Gastroenterology, Gastroenterology Unit, Blackpool Fylde and Wyre Hospitals NHS trust, Whinney Heys Road, Blackpool, Lancashire, FY3 8NR, UK; 2 Department of Radiology, East Lancashire NHS Trust, Burnley General, Hospital, Casterton Avenue, Burnley, BB10 2PQ, UK

## Abstract

**Introduction:**

A congenitally absent Inferior Vena Cava (IVC) is a rare anomaly that is recognised to be associated with idiopathic Deep Venous Thrombosis (DVT), particularly in the young. It may not be apparent until later in life. Retrospectively, as discussed in this case, there can be clues indicating the presence of such an anomaly from a young age. However, it is not clear whether early recognition of this condition would affect the prognosis and treatment.

**Case presentation:**

A 54 year old gentleman was admitted with 3 weeks of abdominal pain and localised swelling over the right flank. Examination revealed palpable 'snake-like' tortuous, tender lumps on the abdominal wall. Past history revealed chronic non-healing venous leg ulcers, and varicose veins necessitating varicose vein ligation at a very young age. The ulcers eventually needed skin grafting.

During this, current admission he was investigated and diagnosed with Deep Vein Thrombosis (DVT). CT scan, performed to search for intra-abdominal cancer, revealed absence of the Inferior Vena Cava with extensive thrombosed collaterals of the superficial abdominal and azygous veins and a congenitally atrophic left kidney.

**Conclusion:**

This is a case of one of the oldest patient described in the literature to be diagnosed with absence of the IVC. It is thought that IVC anomalies are under-diagnosed, and may be commoner than once believed. However there were vital clues in his previous medical history suspicious for an underlying venous anomaly. Idiopathic DVT in a relatively young person with a past history of chronic leg ulceration or varicose veins should be investigated for congenital anomalies of the IVC. This is best achieved by CT scan of the abdomen.

## Introduction

Anomalies of the IVC are well described and recognised but are rare causes of DVT. The prevalence of DVT in those above age 40 years is 1 in 1000 [1], and less frequent, with an incidence as low as 1 in 10,000 in those aged 20–40 years.

Causes of DVT are multifactorial and associated with both environmental and genetic factors that alter the coaguability or flow of blood. When the major recognised risk factors for DVT are absent, in the younger age group presenting with DVT, a search for a genetic or congenital cause is sought.

Here, we present a case of a late presentation of DVT with an underlying aetiology of an absent IVC. The patient lived a generally healthy life with a significant past medical history that could have potentially resulted in an earlier diagnosis of his congenital anomaly. Whether this would have altered the initial management or prognosis is open to debate.

## Case presentation

A 54 year old male presented to a busy district general hospital medical emergency department with abdominal pains and swelling over the right flank for a two to three week period. More recently he noticed swelling of his right lower leg and thigh. He was a non-smoker, and there was no significant family history of disease. He had no upper or lower gastrointestinal symptoms. There was no change in weight or appetite. There was no history of cardio-respiratory disease, and his exercise tolerance was not limited. He was not able to volunteer any further information as regards to his past medical history other than that he was under annual review by nephrologists for mild chronic renal impairment, due to an "atrophic left kidney". This was diagnosed by ultrasound of the renal tract. There was no evidence of any other imaging modalities or radiological investigations undertaken to investigate the cause of his atrophic kidney.

On further review of his medical notes it was revealed that he had a troublesome childhood with bilateral Perthe's disease, and non-healing venous ulcers on the medial aspect of his right ankle.

In 1973, aged 21 years, he underwent skin grafting of a non-healing ulcer. One year later he was re-admitted with recurrence of ulcers in the same region and was then noted to have dilated varicose veins and thrombophlebitis that was treated with crepe bandaging for 2 years. Treponemal serology then was negative. In 1977 he had ligation of the dilated varicose vein that was "feeding the ulcerated part of the leg". In 1979 he was discharged from follow up with complete healing of the leg ulcers.

**Figure 1:**
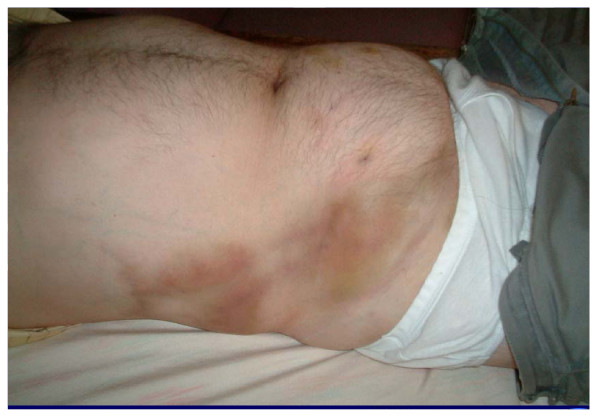
Photo showing dilated superficial abdominal veins (upper quadrant), with bruising and thrombosed large abdominal veins (lower quadrant).

On examination he was noted to have bilateral lower limb varicose veins. Examination of the abdomen revealed large, tortuous, hard, "snake like" palpable veins over the right aspect of his abdomen with overlying bruising. (see photo- figure 1). A preliminary diagnosis of probable Thrombophlebitis Migrans was made. A CT scan was performed to exclude underlying intra-abdominal malignancy.

**Figure 2:**
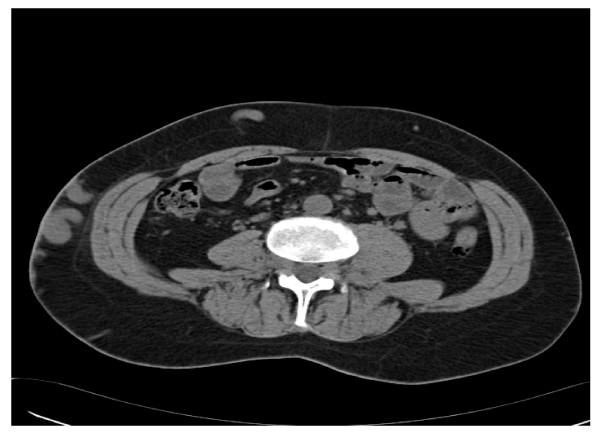
CT abdomen show absent IVC with thrombosis of the very prominent collateral veins in the abdominal wall, corresponding to the right side of the abdomen as seen in figure 1.

The CT scan showed : "A congenitally absent inferior vena cava with collaterals on the anterior abdominal wall and prominent azygous and hemi-azygous veins. Agenesis of the left kidney is noted." (see radiograph- figure 2)

US Doppler revealed : thrombosis in the right iliac and superficial femoral veins.

He was commenced on low molecular weight heparin and warfarin. Low molecular weight heparin was stopped when the International Normalised Ratio (INR) was greater than 2.0.

## Discussion

Absent Inferior Vena Cava (IVC) is an uncommon but well recognised anomaly. Anomalies of the IVC have been described more frequently (0.6%-2%) in those with other cardiovascular defects [2], and less frequently in otherwise healthy individuals. Various anomalies of the IVC have been described including complete absence, partial absence or presence of bilateral IVC [3].

There is controversy as to whether an absent IVC is a true embryonic anomaly or whether it is the result of peri-natal IVC thrombosis causing regression and disappearance of the once present IVC [4].

There has been one previous report in the literature of an absent IVC and left renal hypoplasia- and a right hypertrophic kidney [5]; but a commoner association recognised is right renal aplasia [6] as suggested in a review by Gayer et al, where all nine patients with complete absence of the IVC had an absent or very small right kidney. The association of an absent or hypoplastic kidney is potentially related (or even the cause of an absent IVC) due to peri- atal renal vein thrombosis [7]. It has been proposed by Veen et al [5] to name this condition (when associated with deep vein thrombosis-DVT) KILT (**K**idney and **I**VC abnormalities with **L**eg **T**hromboses) syndrome.

It is estimated that DVT occurs one case per thousand patient-years [8], and in up to 80% a risk factor can be identified. Ruggeri et al [9] presented four cases of absent IVC over a five year period presenting with idiopathic DVT in those below 30 years of age. This was estimated to represent 5% of cases of "idiopathic" DVT in young people.

Chee et al [10] similarly noted that up to 5% of 20–40 year olds presenting with DVT had an IVC anomaly (4 in total- of which 3 had complete absence of IVC). This was much higher than the expected 0.5%.

Both authors concluded that an absent IVC was more common than initially anticipated and may be underestimated or under-reported. Absence of the IVC cannot be detected by ultrasonography, and so a CT scan was recommended in all young patients with an idiopathic DVT.

The ideal imaging modality to diagnose an IVC anomaly must have high diagnostic accuracy, and be safe and reproducible. It is difficult to diagnose any IVC anomaly by ultrasound scanning. Various clues are recognised on radiological imaging that could help diagnose an absent IVC or anomaly. One of the more common and helpful clues is well developed and possibly dilated intra-thoracic hemiazygous and/or azygous continuations. These collateral circulations as well as other retro-peritoneal venous pathways are usually well developed before symptoms present [11].

The most reliable non-invasive methods for diagnosing IVC anomalies are CT with intravenous contrast or Magnetic Resonance scan. CT scan, unlike US, is a good imaging modality of the retro-peritoneal space [12]. Another accurate, but more invasive, imaging modality is venography, which is particularly useful if any surgery is planned.

It is hypothesized that blood return with an absent IVC is inadequate, despite adequate collaterals, resulting in chronic venous hypertension in the lower extremities causing venous stasis which precipitates thrombosis.

Gayer et al [13] recommended that all patients with an IVC anomaly be screened for thrombophilic disorder. In their series 7 of 9 patients with IVC anomaly and DVT had a positive thrombophilic screen.

There have been three case reports in the English language medical literature of thrombo-embolism due to IVC anomaly (absence of the infra-renal portion of the IVC, and Infra-renal IVC hypoplasia). In all of these cases the thrombophilia screen was negative [14-16]. It was hypothesized that multiple emboli from DVT in the common and superficial femoral veins migrate through the well developed hemi-azygous and/or azygous system to the pulmonary circulation.

There is very little evidence available on the surgical correction or treatment of this uncommon anomaly. A case report [7] with complete absence of the IVC but patent iliac veins and non-healing pre-tibial ulceration described successful treatment with a prosthetic graft from the iliac vein to the intra-thoracic azygous vein. Success was defined as complete healing of the ulcer up to 30 months after surgery.

## Conclusion

In conclusion, this gentleman had an extensive past medical history of idiopathic varicose ulceration with evidence of chronic venous hypertension from a very young age. He was managed with difficulty but achieved eventual healing of his ulcers as a young adult. In later life he developed extensive DVT with worsening of his lower limb and abdominal varicosities.

The very limited data from the literature suggests that in cases of an absent IVC in young people (some data below 30 years, other data 20–40 years) the patient should have a CT of the abdomen.

In this case, with a relevant and extensive past history, review of the limited literature would support further radiological investigations to exclude an intra-abdominal deep venous anomaly.

It is unlikely that surgical correction has any role in the management of this patient in the long-term especially many years after healing of his ulcers. There is no consensus regarding the duration of anti-coagulation but it would seem sensible for him to remain on life-long anticoagulation given the on-going risk of further DVT and pulmonary embolism, even if his thrombophilia screen is negative.

Knowledge of the association of other anomalies in those with an absent IVC, such as renal atrophy or agenesis can highlight an underlying vascular anomaly, and can themselves be of significant clinical importance. In this way, diagnostic and treatment pitfalls may be avoided.

## Abbreviations

DVT- Deep vein thrombosis; IVC- Inferior Vena Cava; US- Ultrasound; CT- Computed Tomography.

## Competing interests

The author(s) declare that they have no competing interests.

## Authors' contributions

EN diagnosed the anomaly and supplied the radiographs. JI and EN contributed in preparation of the manuscript. All authors read and approved the final manuscript.

## Consent

Written and informed consent was obtained from the patient for publication of this case report and accompanying images. A copy of the written consent is available for review by the Editor-in-Chief of this journal.
